# Efficacy of multivalent, modified- live virus (MLV) vaccines administered to early weaned beef calves subsequently challenged with virulent *Bovine viral diarrhea virus* type 2

**DOI:** 10.1186/s12917-015-0342-8

**Published:** 2015-02-10

**Authors:** Manuel F Chamorro, Paul H Walz, Thomas Passler, Edzard van Santen, Julie Gard, Soren P Rodning, Kay P Riddell, Patricia K Galik, Yijing Zhang

**Affiliations:** Department of Clinical Sciences, College of Veterinary Medicine, Auburn University, Auburn, AL USA; Department of Pathobiology, College of Veterinary Medicine, Auburn University, Auburn, AL USA; Department of Animal Sciences, College of Agriculture, Auburn University, Auburn, AL USA; Department of Crop, Soils, and Environmental Sciences, College of Agriculture and Alabama Agricultural Experiment Station, Auburn University, Auburn, AL USA

**Keywords:** Early weaning, BVDV, MLV, Vaccine, Antibody titres, Virus, Isolation, Shedding

## Abstract

**Background:**

Vaccination of young calves against *Bovine viral diarrhea virus* (BVDV) is desirable in dairy and beef operations to reduce clinical disease and prevent spread of the virus among cattle. Although protection from clinical disease by multivalent, modified-live virus (MLV) vaccines has been demonstrated, the ability of MLV vaccines to prevent viremia and viral shedding in young calves possessing passive immunity is not known. The purpose of this study was to compare the ability of three different MLV vaccines to prevent clinical disease, viremia, and virus shedding in early weaned beef calves possessing maternal immunity that were vaccinated once at 45 days prior to challenge with virulent BVDV 2.

**Results:**

At 45 days following vaccination, calves that received vaccines B and C had significantly higher BVDV 1 and BVDV 2 serum antibody titers compared with control calves. Serum antibody titers for BVDV 1 and BVDV 2 were not significantly different between control calves and calves that received vaccine D. Following BVDV 2 challenge, a higher proportion of control calves and calves that received vaccine D presented viremia and shed virus compared with calves that received vaccines B and C. Rectal temperatures and clinical scores were not significantly different between groups at any time period. Calves that received vaccines B and C had significantly higher mean body weights at BVDV 2 challenge and at the end of the study compared with control calves.

**Conclusions:**

Moderate to low maternally-derived BVDV antibody levels protected all calves against severe clinical disease after challenge with virulent BVDV 2. Vaccines B and C induced a greater antibody response to BVDV 1 and BVDV 2, and resulted in reduced viremia and virus shedding in vaccinated calves after challenge indicating a greater efficacy in preventing virus transmission and reducing negative effects of viremia.

## Background

*Bovine viral diarrhea virus* (BVDV) is an important cause of respiratory, enteric, and reproductive disease in cattle and has been associated with major economic losses in cattle operations worldwide [1]. Vaccination of young calves against BVDV reduces the number of acute infections in the herd and limits spread of virus among cattle populations [1,2]; however, effective vaccination of young calves against BVDV can be challenging due to the presence of maternally-derived BVDV antibodies at the time of vaccination [3]. Although maternally-derived BVDV antibodies can provide protection against acute BVDV infection and clinical disease, humoral immune responses to vaccination might be adversely affected [4]. Concentration of maternally-derived BVDV antibodies and age of calf at the time of vaccination are important factors in the induction of adequate immune responses following BVDV immunization [5-7]. Calves with moderate to high maternally-derived BVDV antibody levels at vaccination do not usually respond with an increase in BVDV antibodies but are protected against clinical disease, have a slower decay rate of maternal immunity, and develop anamnestic antibody responses following BVDV challenge [8-10]. Calves with low maternally-derived BVDV antibody levels respond to vaccination by increasing BVDV antibody titers. The priming of naive B and T cells, the induction of specific cell mediated immune memory responses, and the induction of anamnestic antibody responses have been identified as the main source of protection of young calves vaccinated in the presence of maternally-derived antibodies and subsequently challenged with virulent BVDV [11-15].

Early weaned beef calves possess variable levels of maternally-derived BVDV antibodies at 2–4 months of age and therefore could benefit from vaccination prior to stress of weaning and shipment. A single dose of a multivalent, MLV BVDV vaccine was demonstrated to be effective in protecting young calves possessing different levels of maternal immunity against acute BVDV infection [16]. In addition to prevention of clinical disease, vaccination should limit the spread of BVDV by reducing virus shedding and horizontal transmission, a desirable outcome of vaccination in herds or units where populations of highly stressed cattle are commingled. However, experimental studies comparing different multivalent MLV vaccines containing BVDV in their ability to prevent viremia and viral shedding in young calves possessing maternally-derived immunity that subsequently undergo challenge with virulent BVDV are limited. The objective of this study was to evaluate the ability of three different commercially available, multivalent MLV vaccines containing BVDV to prevent clinical disease and reduce shedding of virus when administered to early weaned beef calves subsequently challenged with virulent BVDV 2 at 45 days after vaccination.

## Results

Four groups of early weaned beef calves were vaccinated (B, C, and D) or received phosphate saline (A) at weaning at a median calf age of 72.2 days. The calves were born from cows that had been previously vaccinated at least once during the last 2 years with a MLV vaccine. The vaccine used in the cows during the previous 2 years was the same vaccine used in experimental calves from group D. Forty five days after weaning calves were challenged with BVDV 2 1373. Evaluation of clinical responses to challenge and sample collection was performed from day 0 (challenge day) until day 28.

### Serum virus neutralization titers

Thirty days prior to vaccination (day −75) and at a median calf age of 44 days, the mean levels of maternally-derived BVDV 1 NADL and BVDV 2 125c serum antibodies were similar between groups (Table 1). At vaccination (day −45), a significant effect of time (day) was detected (P = 0.0001) as decay of maternally-derived antibodies for BVDV 1 NADL and BVDV 2 125c occurred in all groups; however, mean levels of BVDV 1 and BVDV 2 antibodies were not significantly different (P > 0.05) between groups. Forty-five days after vaccination, which corresponded to the time of BVDV 2 challenge (day 0), a significant effect of group and time (day) was detected (P = 0.0001 and P = 0.0001, respectively). Groups B and C calves had mean levels of serum BVDV 1 NADL antibodies significantly greater than controls (P = 0.0033 and P = 0.0002, respectively). Additionally, on day 0, mean levels of BVDV 1 NADL antibodies were similar between group D and the control group (P = 0.2641). With respect to BVDV 2 125c, group C calves had mean levels of antibodies significantly greater than the control group (P = 0.0368). Twenty eight days following challenge, a significant effect of time (day) was detected (p = 0.0001) with respect to the levels of BVDV 2 125c. The mean BVDV 2 125c serum antibody levels increased similarly in all groups (P > 0.05); in contrast, time (day) and group effects were significant (P = 0.0001 and P = 0.0001, respectively) for BVDV 1 NADL mean antibody levels as these were higher in group B and C calves compared to the control group (P < 0.00001). When antibodies to the challenge strain BVDV 2 1373 were examined at day 0, a significant effect of group and time (day) was detected (P = 0.0001 and P = 0.0001). Calves from groups B and C had greater levels of antibodies compared with control calves (P = 0.05 and P = 0.025, respectively). At day 28 after challenge, a significant (P = 0.0001) effect of time (day) was detected as all groups had increased and similar levels of antibodies to the challenge BVDV strain (P > 0.05).

**Table 1:** **Geometric mean (95% CI) of virus neutralizing serum antibody titers to BVDV 1, BVDV 2, and BVDV 2 1373 from vaccinated (B, C, and D) and unvaccinated (A) calves at each time period**

		**Day of Study**			
**Virus**	**Group**	**−75**	**−45**	**0**	**28**
**BVDV 1 NADL**	A	157.58 (31.12 – 831.74)	60.12 (14.52 – 250.73)	15.03 (5.93 – 38.05)	35.75 (17.38 – 74.02)
	B	135.29^NS^ (28.64 – 639.14)	67.64^NS^ (17.50 – 259.57)	**107.63** ^*^ (62.68 – 183.54)	**861.07** ^*^ (377.41 - 1951)
	C	222.86^NS^ (67.64 – 765.36)	101.12^NS^ (30.27 – 337.79)	**202.25** ^*^ (110.66 – 369.64)	**680.28** ^*^ (225.97 - 2048)
	D	194.01^NS^ (45.56 – 903.88)	95.67^NS^ (22.47 – 407.31)	36.25^NS^ (12.04 – 108.38)	57.70^NS^ (12.99 – 213.78)
**BVDV 2 125c**	A	107.63 (23.10 - 498)	47.83 (13.08 – 173.64)	14.22 (4.08 – 49.18)	608.87 (292.03 – 1260.69)
	B	101.12^NS^ (23.26 – 439.58)	60.12^NS^ (15.67 – 232.32)	42.52^NS^ (22.47 – 80.44)	809^NS^ (455.08 – 1438.15)
	C	128^NS^ (38.85 – 418.76)	85.03^NS^ (26.53 – 272.47)	**60.12** ^*^ (26.35 – 138.14)	1820.34^NS^ (955.42 – 3468.26)
	D	128^NS^ (40.78 – 398.93)	90.50^NS^ (31.34 – 259.57)	29.85^NS^ (15.24 – 58.89)	286.02^NS^ (149.08 – 1541.37)
**BVDV 2 1373**	A	_	_	16.91 (5.02 – 56.44)	861.07 (424.61 – 1734.13)
	B	_	_	**76.10** ^*^ (47.50 – 121.09)	643.59^NS^ (352.13 – 1176.26)
	C	_	_	**93.05** ^*^ (49.18 – 176.06)	1530.72^NS^ (617.37 – 3795.30)
	D	_	_	56.10^NS^ (28.44 – 11.43)	544.95^NS^ (245.57 – 1200.98)

NS, *Means within BVDV strain and Day of Study are not significantly different (NS) from the control group A or are significantly different (*) based on Dunnett’s test at *P* = 0.05.

Among vaccinated groups, seroconversion, as defined as a 4-fold or greater rise in antibody titers to BVDV 1 NADL after vaccination (at day 0) was observed in 16.6% of the calves from group B, 50% of the calves from group C, and 9% of the calves from group D. Similarly, seroconversion to BVDV 2 125c after vaccination was observed in 16.6% of the calves from group B, 8.3% of the calves from group C, and 9% of the calves from group D. None of the calves in group A (control) seroconverted to BVDV 1 NADL or BVDV 2 125c. The proportion of calves that seroconverted to BVDV 2 125c after vaccination was not significantly different between groups (P > 0.05); however, a higher proportion of calves from group C (50%) seroconverted to BVDV 1 NADL with a fourfold increase in antibody titers.

### Virus isolation

**Figure 1:**
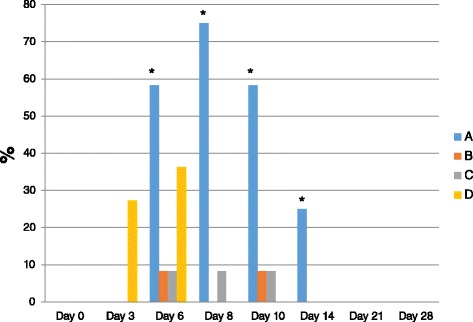
**Total proportion of calves with a positive virus isolation result in WBC and serum samples at each time point after challenge with BVDV 2 1373.** The proportion of vaccinated (B, C and D) and control (A) calves with positive virus isolation WBC or serum samples at each time point (day) after challenge with BVDV 2 1373 was higher and more frequent in the control group (A) compared with groups B and C and D. The star *sign refers to statistical significance (P < 0.05).

Virus positive samples in serum and WBC samples were detected more frequently and for a longer time in the control group compared with groups, B, C, and D (Figure 1). The total proportion of calves that tested positive to BVDV 2 in serum and WBC samples after challenge (days 0 to 28) was higher in the control group (90%) and group D (54.5%) compared with groups B (16%) and C (25%) (Table 2). A significant effect of group and time (day) was detected on days 6, 8, 10, and 14 after challenge with BVDV 2 1373 (P = 0.0001 and P = 0.0001, respectively). On day 6 post-challenge, the control group had a higher proportion of calves with positive BVDV samples (58.3%) compared with groups B (8.3%) and C (8.3%) (P < 0.05) but not with group D (36.3%). Additionally, at days 8, 10, and 14 post-challenge, a higher proportion of calves in the control group had BVDV positive samples compared with calves in groups B, C, and D. The proportion of viremic calves (calves with positive serum or WBC samples) that shed virus after challenge (calves whose nasal swab samples tested positive to BVDV by virus isolation) was higher in the control group (72.7%) compared with groups B (0) and C (0) and D (33.3%) (P < 0.05).

**Table 2:** **Total number and proportion of calves (%) that became viremic and shed virus in nasal secretions after challenge (days 0 to 28) with BVDV 2 1373 in each group**

**Group**	**Number of viremic¥ calves (%)**	**Number of viremic calves that shed virus (%)**
**A**	11 (90)*	8 (72.7)*
**B**	2 (16.6)	0
**C**	3 (25)	0
**D**	6 (54.5)*	2 (33.3)

¥, viremic calves = calves with a BVDV positive sample in WBC or serum.

A higher proportion of control (A) and group D calves became viremic and shed virus after challenge compared with calves from groups B and C. The star *sign refers to statistical significance (P < 0.05).

### Clinical scores and body weight

There were no detectable adverse vaccine reactions in any of the calves. One calf in group D was euthanized on the day prior to challenge with BVDV 2 1373 due to rectal prolapse. Vaccinated and control calves demonstrated clinical protection against challenge with virulent BVDV 2 1373 as only one calf in the control group developed mild diarrhea and anorexia on day 14 post-challenge. The proportion of calves with clinical scores of ≥ 2 for respiratory, diarrhea, and depression parameters was similar between groups (P > 0.05). A mild increase in body temperature, nasal secretion, and loose feces was observed in all calves after challenge independent of group designation. A significant effect of time (day) on rectal temperatures was observed (P < 0.00001). On day 8, the mean rectal temperatures of all groups were increased compared with other days post-challenge.

The average body weight at weaning (day −45) was not significantly different between groups (A = 206.56 +/− 3.53, B = 207.25 +/− 8.33, C = 214.08 +/− 9.25, D = 188.41 +/− 10.08); however, a significant effect of group and time (day) was detected at day 0 and at the end of the study (P = 0.0001 and P = 0.0001, respectively). Calves from group B and C had significantly higher mean body weights at both time points compared with control calves (B = 257.5 +/− 13.14, C = 257.5 +/− 11.87 vs. A = 242.5 +/− 11.08 and B = 345 +/− 15.72, C = 333.75 +/− 12.17 vs. A = 267.91 +/− 12.63, respectively). At the same times (day 0 and end of the trial), the mean body weights of calves from group D were not significantly different compared with control calves (240 +/− 15.25 vs. 242.5 +/− 11.08 and 300.90 +/− 14.7 vs. 267.91 +/− 12.63, respectively) (P = 0.568 and P = 0.262, respectively).

### Hematology

**Figure 2:**
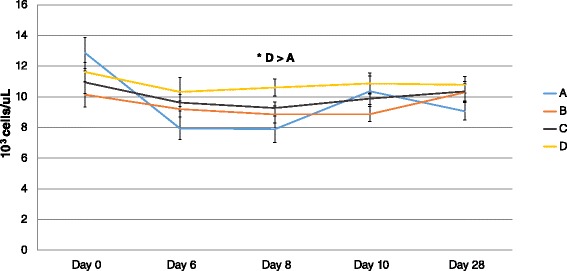
**Mean white blood cell count (+/−SEM) after challenge with BVDV 2 1373.** Mean WBC between vaccinated (B, C, and D) and control (A) calves after challenge. In all calves mean WBC decreased until day 6 after challenge. At day 8 after challenge calves from group D had higher mean WBC compared with the control group (P = 0.007).

**Figure 3:**
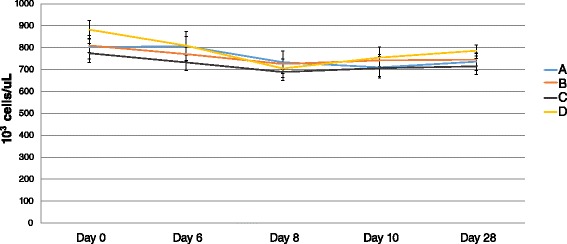
**Mean platelet count (+/−SEM) after challenge with BVDV 2 1373.** Mean platelet count after challenge with BVDV 2 1373 was not significantly different between vaccinated (B, C, and D) and control (A) calves.

A significant effect of time (day) but not group was detected in the mean WBC counts, as mean WBC decreased in all groups from day 0 (challenge) until day 6 post-challenge (P = 0.0001); however, significant differences were not observed between groups (P > 0.05). On day 8 post-challenge, a significant effect of group and time (day) was detected. The mean WBC count from Group D calves was significantly higher compared with control calves (P = 0.007). At the same time, the mean WBC from calves in groups B and C were not significantly different compared to control calves (Figure 2). Effects of group and time (day) were not detected in platelet counts at any time point after challenge with BVDV 1373 (Figure 3).

## Discussion

High levels of BVDV-specific antibodies from colostrum or vaccination effectively protect calves against severe clinical disease induced by challenge with virulent BVDV [3,9]; however, prevention of viremia and virus shedding are variable in cattle vaccinated with MLV vaccines and subsequently challenged with virulent BVDV [17-19]. In the current study, calves from all groups had similar clinical scores and rectal temperatures, and mortality was not observed after challenge with virulent BVDV 2 1373. This could be associated with protection offered by maternally-derived BVDV antibodies in vaccinated and unvaccinated calves as has been previously reported [11], or could have resulted from lower virulence of the challenge virus than in previous reports [20]. Pestiviruses constantly undergo genetic change due to the poor proof-reading capability of the RNA-dependent RNA polymerase, resulting in variability of phenotypic characteristics such as host-cell tropism and virulence [21]. Repeated passage in cell culture was previously reported to result in attenuation of a BVDV isolate, and may have occurred with the BVDV 1373 used in this study [22].

Interestingly, only calves from group D had an increased white blood cell count compared with control calves at day 8 post-challenge. Prevention of leukopenia is one of several parameters used to evaluate response to vaccination after challenge with BVDV [9,11], and in this case the lack of a decrease in WBC counts observed in group D could have been related to the effects of vaccination or to the presence of high levels of specific maternal antibodies. Despite their ability to reduce clinical disease, maternally-derived BVDV antibodies were not as effective in preventing viremia and viral shedding in calves from the control group as 11/12 (90%) of the calves became viremic and of those 8/11 (72.2%) shed virus after challenge. For this study, we chose VI as our testing method in order to document clinically relevant shedding of live virus [23]. Prior to challenge, the geometric mean of serum BVDV 2 1373 antibody titers of control calves was significantly lower compared with titers from groups B and C (16.91 vs. 76.10 and 93.05, respectively). Low serum antibody titers prior to challenge with BVDV have been associated with an increased risk of viremia and clinical disease as demonstrated in previous studies [3,4]. Other reports have indicated that calves with serum maternally-derived BVDV antibody titers < 64 before challenge with virulent BVDV have a higher risk of developing clinical disease and systemic spread of the virus compared to calves with greater antibody titers [3,8].

Antibody response to vaccination with MLV BVDV vaccines of young calves in the presence of maternally-derived BVDV antibodies has produced variable results. Previous studies have demonstrated that 40 to 90-day-old calves with maternally-derived antibody titers ≤ 32 against BVDV 1 and BVDV 2 prior to vaccination seroconvert after vaccination [5-7,10]; additionally, these calves can develop an anamnestic response when a second dose of vaccine is administered [5,10]. In contrast, similar studies have demonstrated that 3 to 56-day-old calves with BVDV 1 and BVDV 2 maternally-derived antibody titers ≥ 32 prior to vaccination usually do not seroconvert to vaccination and clinical protection against challenge with BVDV is variable [4,9,13]. In our study, a small proportion of calves vaccinated at weaning, at a median age of 72.2 days, seroconverted to BVDV 2 after vaccination. In contrast, 50% of calves from group C seroconverted to BVDV 1 suggesting a greater ability of the vaccine C to overcome maternal interference to BVDV 1. The higher levels of antibodies to BVDV 1 NADL and BVDV 2 1373 before challenge, the lower proportion of viremia, and the absence of viral shedding after challenge observed in calves from groups B and C suggests that vaccination with B and C may have primed B cell responses to increase specific antibody production or may have delayed the normal decay of maternal BVDV antibodies [6-8,10]. Additionally, the higher levels of antibodies at challenge could have reduced viremia and prevented viral shedding in calves from the same groups. The reduction of viremia and viral shedding is a highly desirable outcome of vaccination since this could prevent BVDV transmission in operations such as feedlots and stocker units where high numbers of cattle from multiple origins are commingled.

The higher proportion of calves with viremia and virus shedding observed in group D could have been a consequence of the presence of more specific maternal antibodies induced by previous vaccination of the dams with vaccine D. The presence of more specific BVDV antibodies induced by vaccine D on colostra from the dams could have exerted a more efficient blockage of humoral cell responses of calves vaccinated with D. Similar results were detected in a recent study in which lower antibody levels to BVDV 1a, BVDV 1b, and BVDV 2 and a higher proportion of viremia after BVDV 2 challenge were observed in calves 42 days after vaccination with D [19]. Protection against viremia and virus shedding after experimental challenge with BVDV of calves vaccinated with MLV BVDV vaccines has been commonly associated with activation of T cell mediated immune responses independent of the induction of an adequate antibody response [11,13,14]. A previous study reported the depletion of CD4+ lymphocytes in calves acutely infected with BVDV could prolong the duration of viral shedding [24]. Additionally, in another study, 80% of calves vaccinated at 3 days of age with a MLV BVDV vaccine and challenged 7-9 months later with virulent BVDV 2 became viremic after challenge; however, calves were protected against severe clinical disease in the absence of antibody responses at initial vaccination [17]. This indicates that induction of specific T cell memory responses after early vaccination may not always prevent viremia and virus shedding in young calves after challenge with BVDV. In the current study we did not evaluate BVDV-specific T cell responses to vaccination and challenge; however, it is possible that a stronger activation of T cell memory responses in calves vaccinated with D that became viremic could have reduced the duration of viremia to only 2 days; additionally, T cell memory responses in groups B and C could have been associated with the reduced proportion of calves with viremia and nasal shedding.

Previous reports have demonstrated that acute BVDV infection in young calves can result in decreased weight gains and decreased performance [9,11]. In the current study, control calves had lower mean body weights at day 0 and at the end of the study compared with calves from groups B and C. Mean body weights of control and group D calves were similar during the study. It is possible that vaccines B and C had a positive effect on weight gain after vaccination as previously reported [25]. Additionally, the higher frequency of viremia in control and group D calves could have had a negative effect on performance and weight gain. Another study demonstrated that young calves vaccinated with a MLV BVDV vaccine and subsequently challenged with virulent BVDV have higher mean body weights and average daily gains compared with non-vaccinated calves [9]. The higher mean body weights observed in calves from groups B and C at the end of the study suggest that higher levels of BVDV antibodies before challenge and prevention of viremia could have a positive effect on performance, although this observation would need further research using larger numbers of experimental subjects. Prevention of weight loss could be a highly desirable outcome of vaccination programs for early weaned beef calves.

## Conclusions

Vaccination of young calves possessing maternally-derived immunity with multivalent MLV vaccines was demonstrated to be beneficial in reducing viremia and virus shedding following BVDV challenge at 45 days after vaccination. Moderate to low levels of maternally-derived BVDV antibodies protected early weaned beef calves against severe clinical disease induced by challenge with virulent BVDV 2. Decay of maternally-derived BVDV antibodies was observed in all groups and just a small proportion of calves seroconverted to BVDV 2 after vaccination; however, the ability of MLV BVDV vaccines to prime B cell responses, induce antibody production, or delay the decay of maternal immunity could result in reduced numbers of viremic calves and prevent virus shedding as was observed in calves from groups B and C in the present study. Reduction of viremia and BVDV shedding could result in decreased BVDV transmission and disease by increasing calf-herd immunity and reducing environmental load of free virus as has been suggested by a previous study [26]. Additionally, MLV BVDV vaccines that reduce viremia after BVDV challenge could have a positive effect on calf performance and would be of most benefit when establishing health programs for early weaned beef calves.

## Methods

### Animals

Forty-eight crossbred steer calves born and raised at the Upper Coastal Plain Agricultural Research Center, Winfield, AL were utilized in this study. Calves were born in September-October 2012 to cows that had received at least one dose of a modified-live BVDV vaccine D^a^ prior to breeding during the 2 years previous to the start of the study. At birth, calves were identified by an ear tattoo and ear tag. All calves remained with their dams and consumption of colostrum occurred under natural conditions in the pasture. A blood sample for detection of neutralizing antibodies against BVDV 1 and BVDV 2 was collected from all calves at day −75 of the study at a median calf age of 44 days to determine the initial maternally-derived BVDV antibody levels.

### Experimental design

All calves were early weaned on study day −45, which corresponded to calf ages between 62–92 days (2–3 months) with a median age of 72.2 days. To prepare for early weaning, creep feeding was offered for 3 weeks prior to weaning in order to train calves to the weaning diet. The weaning diet was an energy dense (65-75% of total digestible nutrients), relatively high protein (14-17%), and highly palatable ration to meet all nutritional requirements of young growing calves. Calves were stratified by initial maternally-derived BVDV 2 serum antibody titers and assigned by the use of a random number generator ^b^ to 1 of 4 different vaccination groups. The stratification by initial levels of BVDV 2 antibodies ensured that each group received similar numbers of calves with different levels of maternally-derived BVDV 2 antibody levels. All calves underwent abrupt weaning on day −45 of the study and were vaccinated according to their assigned treatment group A (n = 12), B (n = 12), C (n = 12), or D (n = 12). Following vaccination, calves were separated in isolated pastures to prevent transmission of vaccine strains between groups. During this time, daily observation of the calves was performed to evaluate for adverse vaccine reactions. Forty-four days after weaning, calves were transported 192 miles to the North Auburn BVDV Unit located in Auburn, AL. Upon arrival to the North Auburn BVDV Unit, calves were rested and given access to fresh water, hay, and supplement. On the next day (day 0), all calves were challenged with virulent BVDV 2 1373 and placed in the same pasture for the remainder of the study. Clinical evaluation and sampling of calves was performed until day 28 after challenge. All calf protocols were reviewed and approved by the Institutional Animal Care and Use Committee of Auburn University (PRN # 2012–2157).

### Vaccines

All vaccines used were commercially available, USDA-licensed stock material and were administered to calves at weaning (day −45). Group A was the control group and received 2 mL of 0.9% phosphate buffered saline subcutaneously once. Group B received 2 mL of vaccine B^c^ subcutaneously once, group C received 2 mL of vaccine C^d^ subcutaneously once, and group D received 2 mL of vaccine D^a^ subcutaneously once. All vaccines were modified-live and multivalent containing modified-live types 1 and 2 of BVDV, parainfluenza virus 3, bovine herpesvirus 1, and bovine respiratory syncytial virus. All calves in this study were under 6 months of age and vaccination with vaccines B, C, and D was considered off-label.

### BVDV challenge

Forty-five days after vaccination (day 0), all calves were experimentally inoculated with the noncytopathic (NCP) BVDV 2 strain 1373. The NCP BVDV 2 strain 1373 has been previously used in experimental BVDV inoculation of calves and shown to induce severe clinical disease, leukopenia, and thrombocytopenia [27]. The BVDV 2 strain 1373 was propagated in Madin-Darby bovine kidney (MDBK) cells in minimum essential medium ^j^ (MEM), supplemented with 10% equine serum, L-glutamine, penicillin G (100 units/ml), and streptomycin (100 μg/ml). Virus was harvested from cells by a single freeze-thaw method, aliquoted, and stored (−80°C) until needed. Aliquots were enumerated using the method of Reed and Muench [28], prior to inoculation of calves. All calves were inoculated by intranasal aerosol administration of 1 × 10^6^ TCID_50_ of the BVDV 2 strain 1373.

### Sample and data collection

Daily clinical observations were performed by the same person, who was blinded to study group allocation, on days 0, 3, 6, 8, 10, 14, 21, and 28. Additionally, individual rectal temperatures, serum, whole blood, and deep nasal swab samples were collected on those same days. Whole blood samples were subjected to hematologic analysis on day 0 prior to challenge and on days 6, 8, 10, and 28 after challenge for individual white blood cell and platelet counts.

During sampling days, each calf was scored prior to handling for signs of abnormal respiration, diarrhea, and depression using a scale of 0 to 3, with the absence of a clinical sign scored as 0 and the most severe clinical sign scored as 3 [17]. Briefly, an abnormal respiratory score was given if an animal presented with a cough, labored breathing, nasal, or ocular discharge. Nasal and ocular discharges were judged as being serous, mucous, or mucopurulent. Diarrhea scores were judged as being normal feces, pasty feces, runny feces, or severe diarrhea with or without blood. Depression scores ranged from no depression, mild depression, moderate depression, or severe depression. In addition to visual examination, individual body weights were obtained on day −45 (vaccination/weaning day), day 0 (challenge day), and days 14, and 28 after challenge using a portable livestock electric scale ^f^ that was validated prior to and after each weighing.

### Virus Isolation (VI)

Whole blood, serum, and deep nasal swab samples collected on days 0, 3, 6, 8, 10, 14, 21, and 28 after challenge were used for BVDV VI using the immunoperoxidase monolayer assay with techniques previously described [29]. Briefly, the isolated samples were suspended in 24-well plates and subsequently seeded in 50 μL culture medium. The cell suspension was subjected to co-cultivation on 25 cm^3^ flasks containing monolayers of MDBK cells and was incubated for 24 hours at 38.5°C and 5% CO_2_. Following cultivation, 50 μl of the cell culture supernatant was inoculated in triplicate into wells on 96-well microtiter plates containing monolayers of MDBK cells in culture medium. After 96 hours of incubation at 38.5°C and 5% CO_2_, all samples were frozen at −80°C and subsequently thawed to detect BVDV using the immunoperoxidase monolayer assay as previously described [29].

### Virus Neutralization (VN)

The standard virus neutralization microtiter assay was used to detect antibodies against BVDV in serum of calves collected on days −75, −45, 0 (prior to challenge), and 28 [28]. The BVDV 1 cytopathic strain NADL and BVDV 2 cytopathic strain 125c were used. For samples collected on days 0 and 28, the challenge BVDV 2 1373 non-cytopathic strain was also used. Briefly, following heat inactivation at 56°C for 30 minutes, serial 2-fold dilutions (1:2 to 1:4096) were made in 50 μL of culture medium. For each dilution, 3 wells of a 96-well plate were inoculated with an equal volume (50 μL) of culture medium containing 100–500 TCID_50_ of the test strain. After inoculation, the plate was incubated at 38.5°C in a humidified atmosphere of 5% CO_2_ and room air for 1 hour. Then, 2.5 × 10^3^ MDBK cells in 50 μL of culture medium were added to each well. Plates were incubated for 72 hours and evaluated visually for cytopathic effect for the BVDV 1 and 2 cytopathic strains [29,30] or by staining the plates using the immunoperoxidase assay for the BVDV 2 1373 non-cytopathic strain. The geometric mean of antibody titers was calculated from the endpoint Log_2_ titers of the animals in each group. Seronegativity to BVDV 1 and BVDV 2 was defined as a serum antibody titer less than 2 which equates to a Log_2_ antibody titer of 0.

### Statistical analysis

All statistical analyses were performed using the GLIMMIX procedure in the SAS 9.3 software package ^g^. To detect changes in Log_2_ transformed antibody levels, virus isolation, rectal temperatures, white blood cell counts, platelet counts, and body weights, a repeated measures generalized linear mixed model [Response = Group + Time (day) + Group * Day] was performed using an appropriate distribution function. The repeated nature of this experiment, viz. multiple observations on the same experimental unit = animal over time, implies non-independence of residuals. Hence, the residual variance was modelled to arrive at a reasonable residual covariance structure using Akaike’s Information criterion corrected for small sample sizes (AICc) to determine the best structure. A first-order autoregressive structure with heterogeneous variances (ARH 1) was most commonly fitted. This structure allows for a separate residual variance at each time point and a correlation among time points that diminishes with the lag. Because clinical scores for respiratory distress and diarrhea were binary in nature (only scores 1 and 2 were given) the binary distribution function was used in the abovementioned procedure; this analysis approach is commonly referred to as logistic regression. No analysis was performed for clinical depression score as all scores equalled zero. Dunnett’s test for multiple comparisons was used to detect differences between vaccinated groups and the control group; a probability of P ≤ 0.05 was considered statistically significant for all tests. The FREQ procedure in the abovementioned software package ^h^ was used to analyze the proportion of calves with viremia and virus shedding with a *χ*^2^ test.

## Endnotes

^a^Bovi-Shield Gold 5, Zoetis Animal Health, Florham Park, NJ

^b^Microsoft Excel 2010, Redmond, WA

^c^BRD-Shield, Novartis Animal Health, Larchwood, IA

^d^Express 5, Boehringer Ingelheim Vetmedica, Ridgefield, Conn.

^e^Powder River Cattle & Livestock Equipment, Provo, UT

^f^True-test Inc. Mineral Wells, TX

^g^SAS Institute Inc., Cary, NC

^h^PROC FREQ SAS 9.2, SAS Institute Inc., Cary, NC

## Abbreviations

BVDV: Bovine viral diarrhea virus
MLV: Modified- live virus
CP: Cytopathic
NCP: Non-cytopathic
VI: Virus isolation
VN: Virus neutralization
